# 3D Cell Culture for the Study of Microenvironment-Mediated Mechanostimuli to the Cell Nucleus: An Important Step for Cancer Research

**DOI:** 10.3389/fmolb.2021.628386

**Published:** 2021-02-10

**Authors:** Apekshya Chhetri, Joseph V. Rispoli, Sophie A. Lelièvre

**Affiliations:** ^1^ Biomedical Engineering, Purdue University, West Lafayette, IN, United States; ^2^ Department of Basic Medical Sciences, Purdue University, West Lafayette, IN, United States; ^3^ Center for Cancer Research, Purdue University, West Lafayette, IN, United States

**Keywords:** mechanotransduction, mechanosensing, extracellular matrix, microfluidics, tensegrity, epigenome, nucleoskeleton, phenotypic heterogeneity

## Abstract

The discovery that the stiffness of the tumor microenvironment (TME) changes during cancer progression motivated the development of cell culture involving extracellular mechanostimuli, with the intent of identifying mechanotransduction mechanisms that influence cell phenotypes. Collagen I is a main extracellular matrix (ECM) component used to study mechanotransduction in three-dimensional (3D) cell culture. There are also models with interstitial fluid stress that have been mostly focusing on the migration of invasive cells. We argue that a major step for the culture of tumors is to integrate increased ECM stiffness and fluid movement characteristic of the TME. Mechanotransduction is based on the principles of tensegrity and dynamic reciprocity, which requires measuring not only biochemical changes, but also physical changes in cytoplasmic and nuclear compartments. Most techniques available for cellular rheology were developed for a 2D, flat cell culture world, hence hampering studies requiring proper cellular architecture that, itself, depends on 3D tissue organization. New and adapted measuring techniques for 3D cell culture will be worthwhile to study the apparent increase in physical plasticity of cancer cells with disease progression. Finally, evidence of the physical heterogeneity of the TME, in terms of ECM composition and stiffness and of fluid flow, calls for the investigation of its impact on the cellular heterogeneity proposed to control tumor phenotypes. Reproducing, measuring and controlling TME heterogeneity should stimulate collaborative efforts between biologists and engineers. Studying cancers in well-tuned 3D cell culture platforms is paramount to bring mechanomedicine into the realm of oncology.

## Introduction

Force variations at the cellular level are a source of biological modifications that influence organ development and homeostasis (Eckes and Krieg, 2004; Mammoto and Ingber, 2010; Humphrey et al., 2014; Schroer and Merryman, 2015; Barnes et al., 2017). The extracellular matrix (ECM), the protein network of which connects the different parts of an organ and belongs to the cells’ microenvironment, is considered to regulate and propagate mechanical forces (Frantz et al., 2010).

The central role of the ECM in tissue homeostasis had been suggested early on and motivated its inclusion in cell-based research (Bissell, 1981; Ingber et al., 1981). It was the birth of three-dimensional (3D) cell culture, for which the organization of cells into recognizable tissue structures, whether normal or sickly, is paramount. The tumor microenvironment (TME) has been extensively studied with 3D cell culture models. It encompasses noncancerous cells (fibroblasts, endothelial cells, epithelial cells, immune cells like macrophages and dendritic cells) and molecules that sculpt the ECM (e.g., collagen, laminin, elastin, fibronectin, matrix metalloproteinases, elastases, cathepsins). The type and amount of TME components are characteristics of each specific form of cancer (Balkwill et al., 2012; Lu et al., 2012; Hirata and Sahai, 2017). Importantly, dynamic remodeling of the TME associated with stiffening favors aggressive cancer phenotypes (Cheng et al., 2009; Zhao et al., 2014). For instance, increased collagen I deposition and stiffer stroma distinguish aggressive (Basal-like; Her2) from less aggressive (Luminal A and B) breast cancer subtypes (Acerbi et al., 2015). Thus, exploring, *in vitro*, the mechanisms of ECM-cell interactions that control phenotypes requires 3D cell culture.

The concept of tensegrity states that cells are in an active prestress condition secured by a cable-like physical linkage between the ECM and cytoskeletal proteins (Ingber, 1993). Such condition stabilizes and counterbalances forces between intracellular and extracellular compartments. This concept was initially demonstrated by the stiffening response transmitted to the cytoskeleton as a result of mechanical stress directly applied to integrins (Wang et al., 1993). The mammalian cell nucleus was also shown to react to mechanical stressors in the microenvironment (McGregor et al., 2016; Stephens et al., 2019). Reciprocally, cells exert traction on the ECM, as it was shown through deformations on silicone and collagen substrates by chicken fibroblasts (Harris et al., 1980). Constant communication between the ECM and the cells may be viewed as dynamic reciprocity (Bissell et al., 1982), a theory originally substantiated by results from Ingber and Bissell laboratories (Maniotis et al., 1997; Lelièvre et al., 1998). Dynamic reciprocity outlines a model for force-mediated interactions between the ECM and the cell nucleus via transmembrane proteins, cytoskeletal components, centrioles involved in the regulation of cell division, and nuclear components, including the genome, to ultimately affect gene transcription; conversely, changes in gene expression could modify the composition of the ECM.

The conversion of physical forces into biochemical signals, termed mechanotransduction, has been considered a major structure-function relation in cells ultimately leading to a biological outcome (Watson, 1991). The opening of membrane ion channels in response to stretch provided an early demonstration of cellular mechanotransduction (Craelius et al., 1988). Since then, standard 2D culture has confirmed the existence of intracellular mediators of forces, including cytoskeletal elements (e.g., vimentin, talin, microfilaments, microtubules, intermediate filaments) (Liu et al., 2015; Tapia-Rojo et al., 2020), and at the level of the nuclear envelope, linker of nucleoskeleton and cytoskeleton (LINC) complexes and lamins (Buxboim et al., 2014; Guilluy et al., 2014; Janota et al., 2020). In the cell nucleus, mechanotransduction studies are complicated by an organization exquisitely determined by tissue architecture (Chandramouly et al., 2007; Lelièvre and Chittiboyina, 2018), which requires using 3D cell culture to recapitulate the assembly and phenotype of cells as *in vivo*.

In this article, we are placing cell culture models in perspective to illustrate how mechanotransduction to the cell nucleus may be studied beyond standard 2D culture. Microenvironmental forces have been focused on ECM network remodeling, notably in cancer. Yet, current considerations of fluid flow in the microenvironment should provide critical additional information on the impact of TME-mediated mechanical forces on phenotypes (Rothbauer et al., 2018). Cell culture models that integrate ECM and microfluidics to study cancer progression via an influence on the phenotypic heterogeneity of cancers are discussed to highlight how they might feed information necessary for the development of mechanomedicine.

## Evidence of Mechanotransduction to the Genome in Cell Culture

**FIGURE 1:**
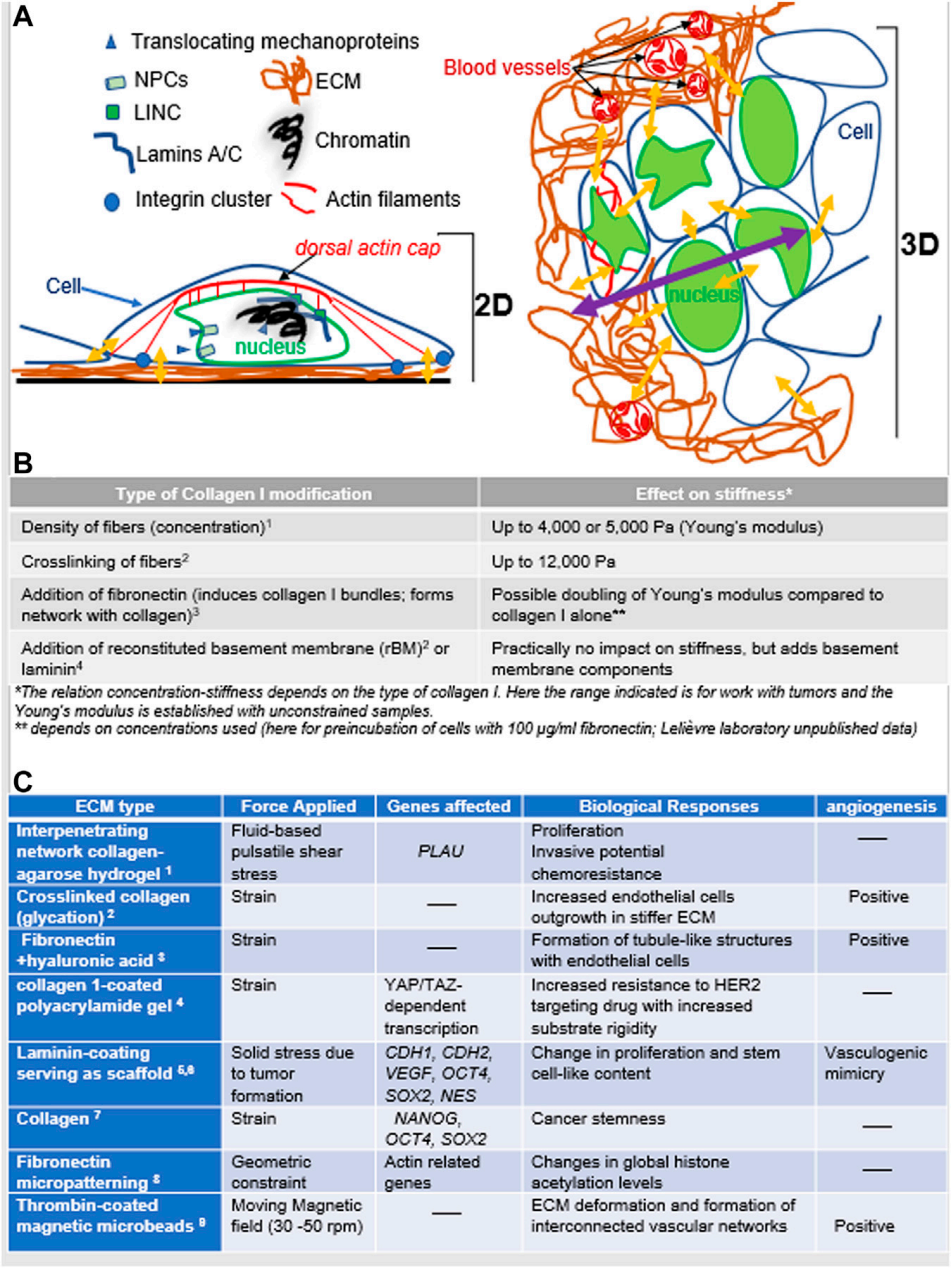
Mechanotransduction in response of mechanostimuli **(A)**. *Left*: In 2D culture, cancer cells deposit their own ECM on the plastic surface, with focal adhesion (integrin clusters) on the ECM against the hard culture surface; there are also possible tension forces between cells. Two interacting modes of mechanotransduction include 1) the translocation of mechanoproteins (YAP/TAZ, myocardin-related transcription factor; MRTF; muscle LIM protein, MLP, etc.) upon cytoskeletal rearrangement that influence gene transcription and 2) the balance of forces between cytoskeleton and the network of lamins and other nuclear proteins via the LINC complexes, ultimately influencing chromatin compaction and gene transcription. *Right*: In 3D culture, the organization of cancer cells into a tumor changes not only the type of forces involved but also the intracellular architecture (e.g., different organization of actin microfilaments and no more dorsal actin cap; great variations in nuclear morphometry depending on the epigenome of cells and their location in the tumor). There are variations in the forces received if cells are at the periphery or deep within the tumor. Yellow arrows indicate a sample of areas of mechanostimuli (the impact from the cell culture medium is not included); the purple arrow indicates increasing distances between cells inside the tumor (with multilayering of cells) and the bulk of the ECM that might create heterogeneity in mechanostimuli. Finally, matrix stiffening also increases angiogenesis via an influence on endothelial cells. **(B)**. Examples of ECM stiffness tuning based on collagen I (^1^ Chhetri et al., 2019, ^2^ Levental et al., 2009, ^3^ Paten et al., 2019, ^4^ Chittiboyina et al., 2018). Other matrices of nonmammalian origin may also be used (e.g., agar, alginate, polyacrylamide) and are sometime mixed with ECM molecules (e.g., collagen I, fibronectin). **(C)**. Cell culture-based examples of the impact of different types of matrices and forces that result in changes in gene transcription and angiogenesis as they relate to cancer aggressiveness (^1^ Novak et al., 2019, ^2^ Bordeleau et al., 2017, ^3^ Seidlits et al., 2011, ^4^ Lin et al., 2015, ^5^ Larson et al., 2014, ^6^ Brodaczewska et al., 2019, ^7^ Pankova et al., 2019, ^8^ Jain et al., 2013, ^9^ Sewell-Loftin et al., 2017).

The transfer of mechanostimuli from the microenvironment to the genes encompasses two major modes, the coupling protein complexes, like LINC, that bridge cytoskeleton and nucleoskeleton, and nuclear pore complexes (NPC) that control the passage of signaling molecules above 35 kDa. Proteins that translocate to the cell nucleus through NPCs in response to mechanostimuli are mechanosensors if they react to cytoskeletal rearrangement when the ECM stiffens or their expression increases on rigid substrates (Moreno-Vicente et al., 2018). They are mechanotransducers via their interaction with transcription factors in the cell nucleus, leading to changes in gene transcription (Foster et al., 2017; Sidorenko and Vartiainen, 2019; Pocaterra and RomaniDupont, 2020). Here, mechanotransduction to the genes may be compared to delivery modes for which mechanical forces are converted into biochemical signals in the cytoplasm (Figure 1A).

Mechanotransduction via LINC is based on balancing intracellular tension. The structural proteins SUN and Nesprin connect actin microfilaments and the outer nuclear envelope (Stewart-Hutchinson et al., 2008). Then, the propagation of mechanical forces to the genome might include structural proteins that span the nuclear interior and form small networks and/or organize chromatin, like nuclear actins (Plessner et al., 2015) and lamins A/C. These and other fibrous proteins along with ribonucleoproteins shape the scaffold of the nucleus that is compared to a nucleoskeleton (Pederson, 2000). The lamins regulate the organization of the genome via an influence on chromatin binding to the nuclear envelope (Makatsori et al., 2004); lamins A/C expression varies depending on ECM stiffness, with resulting effects on gene transcription and phenotypic differentiation (Swift et al., 2013). The cytoskeletal network influences protein translocation and nuclear stiffness (itself linked to the nucleoskeleton and chromatin) (Janota et al., 2020). The nucleus is 2-10 times stiffer than the cytoplasm; yet, it is dynamic and its envelope is in direct contact with chromatin, allowing for an influence on genome architecture and the translation of physical signals into biochemical changes (Uhler and Shivashankar, 2017) (Figure 1A). The direct connection between integrins, the cytoskeleton, chromatin stretching, and the expression of a GFP-tag reporter gene was elegantly demonstrated using magnetic twisting cytometry (Tajik et al., 2016). Moreover, transcriptional modifications associated with the application of mechanical forces via integrins involve changes in H3K9 methylation that depend on the location within the cell nucleus (Sun et al., 2020).

Many experiments on mechanosensing and mechanotransduction did not reproduce the tissue context for which cell shape and intracellular organization are essential characteristics. Yet, the nucleus can acquire specific roles within a 3D collagen I gel, as shown by its control of contractility, in contrast to cells cultured on top of the gel (Graham et al., 2018). The importance of 3D cell culture to study the nucleus was initially demonstrated via the dynamic distribution of the nuclear structural protein NuMA that responds to ECM signaling (Lelièvre, et al., 1998; Vidi et al., 2012), and itself controls phenotypically normal differentiation via an action on the epigenome (Abad et al., 2007). Epigenetic organization also depends on tissue architecture (Plachot and Lelièvre, 2004; Chandramouly et al., 2007), reinforcing the value of 3D cell culture to unravel the influence of mechanotransduction to the cell nucleus on phenotypes. As an encouraging step towards ECM-based cell culture to study mechanotransduction to the epigenome, deformation of mouse oligodendrocytes seeded on stretchable silicone rubber-coated with Matrigel revealed the involvement of SYNE1, a component of LINC, in increased expression of the epigenetic silencing marker H3K9me3 (Hernandez et al., 2016). For the epigenetic disorder that is cancer, it is essential to study mechanotransduction in tumor models in 3D cell culture.

## Cell Culture Models for Extracellular Matrix-Mediated Mechanical Forces in Cancer Progression

The discovery that tumors are stiffer than healthy tissues has encouraged the study of mechanotransduction in 3D cell culture. Overall, models employing collagen I-based control of ECM stiffness have demonstrated that mechanotransduction might play an essential role in phenotypic switches throughout the neoplastic process, from an ‘at risk’ phenotype to an invasive cancer phenotype. For instance, increasing collagen I density to augment stiffness by a factor of 2 altered normal mammary morphogenesis, as evidenced by loss of polarity, although basement membrane components that are important to maintain differentiation were also included (Paszek et al., 2005). It was accompanied with the activation of Rho kinase, a regulator of the cytoskeleton known to respond to mechanical stress (Wojciak-Stothard and Ridley, 2003). Noticeably, increased collagen density is considered an aggravating factor of breast cancer risk (Turashvili et al., 2009), and loss of polarity is necessary for cancer onset as shown in 3D cell culture (Chandramouly et al., 2007; Bazzoun et al., 2019). Stiffening the ECM via collagen I cross-linking also disrupted epithelial organization, and in combination with oncogenes, drove invasive behavior via integrin clustering (Levental et al., 2009). Stiffening may be further increased by collagen I and fibronectin interaction; however, the organization of the ECM also depends on cellular traction forces (Kubow et al., 2015), which nicely illustrates the dynamic reciprocity concept. Mechanotransduction equally occurs in the stromal cells that secrete interstitial ECM and influence cancer development, as shown by the involvement of focal adhesion kinase (FAK) in fibroblast migration through a dense collagen I matrix (Mierke et al., 2017).

Importantly, mechanotransduction in tumor cells may be induced not only through cell-ECM interaction but also by cell-cell interaction. Cell-mediated mechanical stress has been measured by cell-sized oil microdroplets with defined mechanical and adhesion properties introduced between cells (Campàs et al., 2014). Mechanotransduction induced by an increased matrix stiffness also influences endothelial cells. Using 3D matrices made of collagen, it was shown that a stiffer ECM promoted by glycation (but not a denser matrix) increased angiogenesis via the upregulation of matrix metalloproteinases and that the stiffness corresponding to a TME altered the integrity of the endothelial barrier as *in vivo* (Bordeleau et al., 2017) (Figure 1A). For details we refer the readers to a well-documented summary of the literature on the impact of mechanical forces on tumor angiogenesis, notably highlighting the mechanosensory complexes activated in response to mechanical forces in endothelial cells (Zanotelli and Reinhart-King, 2018).

A simple initial approach to introduce ECM stiffness in 3D cell culture is to use collagen I hydrogel that can be fine-tuned to a selected Young’s modulus (Chhetri et al., 2019) and mixed with other ECM molecules to further alter stiffness or stimulate diverse biochemical signaling pathways (Figure 1B). However, setting 3D cell culture conditions requires information on *in vivo* stromal Young’s modulus. For instance, a parallel increase in collagen deposition (Trichome staining) and matrix stiffness (atomic force microscopy-AFM) was observed from healthy to cancerous preinvasive and cancerous invasive human breast tissues, with a 4-5 fold stiffness increase for the latter corresponding to a Young’s modulus of 3,000 Pa on average based on unconfined compression analysis (Acerbi et al. 2015). It is important to understand that not only the stiffness of the matrix, but also its type play critical roles in 3D cell culture. For instance, using solely a fibronectin network has been shown to promote epithelial to mesenchymal transition (Jordahl et al., 2019) and the addition of hyaluronic acid to fibronectin stimulates angiogenesis (Seidlits et al., 2011). Reproducing a TME characteristic of specific types of cancer is especially important to study cancer therapies, as shown with the production of different types of HA-rich ECM emulating the brain parenchyma (Blehm et al., 2015). Stabilizing the high stiffness level of collagen I and fibronectin necessary for 3D culture of tumors may be achieved with photocrosslinking (Seidlits et al., 2011; Nguyen et al., 2019). Synthetic polyhydrogels are also being developed for use in 3D cell culture. They can be crosslinked physically (ionic/H-bonding/hydrophobic forces) or chemically by covalent process in order to provide the degree of elasticity necessary for a TME. The reversibility and thus, poor mechanical properties of physically crosslinked polymers is their major limitation affecting the overall stiffness of the matrix (Parhi, 2017). Examples of synthetic hydrogels usable for tumor culture include Polyethylene glycol (PEG) and Polycarpolactone (PCL) that can be crosslinked chemically and provide stiffness conditions within the wide range of mechanical properties of many tumors (0.4-10 kPa). However, these hydrogels lack the essence of biological signaling unless they are functionalized, for instance by adding peptide sequences of ECM components. The great capabilities for architectural modeling or patterning and for functionalization of these types of matrix usually leads to highly specialized uses like therapeutic approaches (e.g., for use *in vivo* for drug delivery), controlled matrix degradation, study of migration in complex matrix densities, cancer stem cell enrichment (Singh et al. 2014; Palomeras et al., 2016).

Rheology (i.e., the deformation and flow of materials that give viscoelastic information) governs physical cellular responses to mechanical stress that occur over time (Bonfanti et al., 2020), and tensegrity is one of the rheological models (Van Citters et al., 2006). The complexity of cellular mechanical properties lies in part on different reactions depending on cortical and deep locations and requires various models and advanced measurement tools like particle-tracking microrheology, optical tweezers, AFM, traction force microscopy, magnetic bead cytometry, optical stretchers, micropipette aspiration and microplate rheometer. Unfortunately, studying rheology in 3D cell culture is short of a magical process, since most measurement and mathematical tools have been established with standard 2D cell culture and do not accommodate for the thickness and depth of tumors. Importantly, 2D cell culture on plastic artificially increases stiffness (via “bottom effects”) measured with AFM indentation; 3D culture that can be done on top of ECM for certain tissues provides greatly improved measurement conditions (Guimarães et al. 2020). An optical trap that senses thermal fluctuations of lipid granules was used to compare the intracellular viscoelastic properties of invasive breast, colon and pancreatic cancer cells with noninvasive cells cultured in low (1 mg/ml) and increased (4 mg/ml) collagen I density in 3D culture of tumor nodules. A statistically significant adjustment in cellular viscoelasticity was observed within 24 h of exposure to increased ECM stiffness, but only in the invasive cells and with increased viscosity at the invasive edge (Wullkopf et al., 2018).

Even more difficult is to apprehend the physics of the cell nucleus. Magnetic tweezers that nicely revealed the involvement of nuclear lamins in nucleus resistance to shear forces are not an easy option for 3D cell culture (Guilluy et al., 2014). In tumors, we have used nuclear morphometry as evidence of a physical impact of increased collagen I stiffness (Chittiboyina et al., 2018). There was a significant nuclear deformation (via a decrease in circularity) when comparing 1500 to 800 Pa for the ECM. Nuclear deformation appears to influence the ATR protein that controls chromatin association to the nuclear envelope, as shown by cell stretching in 2D culture, which might provide a means for the genome to cope with mechanical stress (Kumar et al., 2014). As further evidence of an impact of mechanical forces on the cell nucleus we have included examples of alterations in gene expression depending on the type of ECM and forces (Figure 1C).

## Integration of Microfluidics in the Study of Mechanotransduction in Tumors

*In vivo*, cell nutrition and oxygenation rely on fluid extravasation from blood vessels. In tumors, cells are subjected to strong solid and shear stresses. Rapid tumor growth contributes to solid stress, which in turn, subjects the TME to both tensile and compressive stresses and increases interstitial fluid pressure (Griffon-Etienne et al., 1999). Interstitial flow causes shear stress ranging from pulsatile and turbulent (near capillaries) to primarily laminar convection, with fluid velocity influenced by interstitial porosity and pressure as well as capillary density, permeability, and viscoelastic properties (Follain et al., 2020). In breast tumors, where local vascularization is highly modified, with leaky vessels for instance, fluid flow is increased approximately five-fold in the interstitium compared to normal tissue and results in higher hydrostatic pressure (Butler and Grantham, 1975). Dynamic contrast-enhanced magnetic resonance imaging of murine xenografts of primary tumors has revealed higher interstitial fluid pressure in metastatic compared to nonmetastatic cancers (Hompland et al., 2012).

Cell culture platforms engineered with microfluidic channels are revealing that fluid movement is an important contributor to the mechanostimulation that influences tissue phenotypes. They may be single or multi-chambered and are built with biocompatible substrata, like the popular silicon-based organic polymer polydimethylsiloxane (PDMS). In addition to the speed of delivery into, and retrieval from the platform, the width and depth of the microchannels contribute to the control of shear stress (Cioffi et al. 2010). Since the interstitial fluid goes through a matrix that influences diffusion depending on the density of ECM fibers, mechanotransduction experiments should integrate information from both fluid movement and ECM stiffness; in such case, it is valuable to integrate stiffness biosensors within the cell culture platform (Zareei et al., 2020). Specialized microfluidic platforms, like the gradient-on-a-chip, that generate gradients of molecules in the ECM permit the identification of thresholds for their action depending on ECM stiffness and fluid movements, hence combining physical and chemical stimuli in the microenvironment (Chittiboyina et al., 2018).

Fluid impact in cancer research *in vitro* has been mostly studied in the context of cell motion, a necessary precursor to metastasis. For instance, physiological levels of fluid shear stress (0.1-0.75 dynes/cm^2^ for flow rate 3.9-26 µl/min) experienced by glioma cells embedded in 2 mg/ml collagen I in a modified Boyden chamber, prevented migratory and invasive capabilities for some of the cell types (Qazi et al., 2011). High speed (4.6 μm/s) of interstitial fluid was shown to control the direction of migration of breast invasive tumor cells embedded in 2 mg/ml collagen I gel towards regions of high fluid pressure (that would normally correspond to leaky blood vessels) by influencing asymmetric cell-matrix adhesion and the location of cytoskeletal molecules (Polacheck et al., 2014). Biological responses included standard mechanotransduction pathways, with activation of integrins and autophosphorylation of FAK. Other studies attempted to mimic shear stress in a vessel for metastatic cells. Translocation of YAP, in response to fluid wall shear stress, was associated with the control of genes that promote metastasis (Lee et al., 2017); whereas the translocation of TAZ seemed to control cell proliferation (Lee et al., 2018).

**FIGURE 2:**
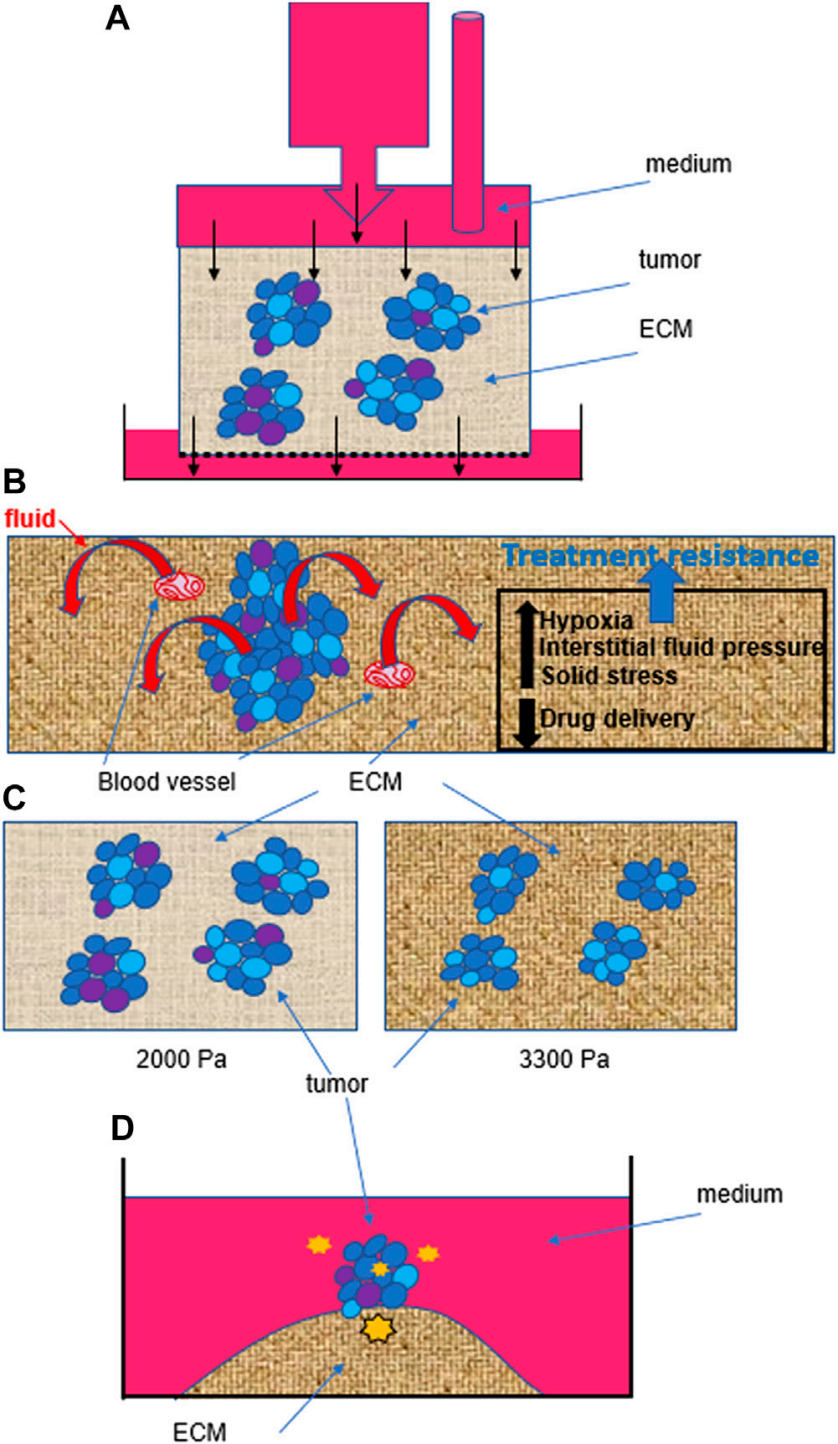
Heterogeneity within and outside tumors influences cancer behavior. **(A)**. Example of fluid stress induced by hydrostatic pressure on isolated cells (Qazi et al. 2011), but that might be applied to tumors in 3D culture. Even if the hydrostatic pressure is homogenous at the top of the device, the phenotypic heterogeneity within tumors (represented by different colors of cells) as well as the heterogeneity in the size of pores (0.1–30 microns) in the ECM are likely to induce different cell responses and thus, behaviors of tumors. Black arrows indicate flow direction. **(B)**. Solid stress linked to tumor growth will also create intratumor heterogeneity by influencing intratumor pressure which pushes fluid out of the tumor, and increasing interstitial fluid pressure, which contributes to hypoxia in different regions of the tumor. Moreover, solid stress and increased matrix stiffness also lead to the compression of blood vessels. Altogether, intratumor heterogeneity, hypoxia and a decreased efficacy in drug delivery (due to blood vessel compression and increased interstitial fluid pressure) contribute to treatment resistance. **(C)**. There might also be selective pressure from mechanostimuli, simply based on an increased matrix stiffness, that would modify intratumor heterogeneity by inducing cell death, as we measured when culturing triple negative breast cancer T4-2 cells for 10 days in 2000 Pa compared to 3300 Pa collagen I matrix (% increase between 25 and 50%, depending on the biological replicate; *n* = 3). **(D)**. Even when starting from one individual cell, without purposefully inducing a mechanostimulus, the tumor that forms on an island of collagen I (3300 Pa) presents cellular heterogeneity (Jain et al., 2020). We propose that in addition to inherent genetic instability of cancer cells during division, as the tumor develops, cells are experiencing different degrees of mechanical forces (represented here by orange stars of various sizes) from within and outside the tumor, depending on their location. Such heterogeneity in mechanostimuli contributes to different levels of mechanotransduction to the cell nucleus and thus, differential gene transcription and phenotypic switch.

For work on primary tumors, fluid-mediated mechanical impact will be best investigated in 3D cell culture, with tumors grown with appropriate ECM stiffness, since high interstitial fluid pressure may drive fluid efflux from the tumor core, hence inducing fluid stress within tumors too. Systems based on the generation of hydrostatic pressure might be applicable to whole tumors (Figure 2A). The study of fluid impact on a tumor will require measuring global tumor deformation as well as intracellular modifications. The study of solid stress performed with tumors grown in agarose matrices, in which confining environments might limit tumor growth and increase cellular packing density (Helmlinger et al., 1997), has revealed that tumor cell size might be used as a measurement of solid stress inside a tumor (Roose et al., 2003). Such architectural response would result from the combination of pressure from the TME, cell-cell interactions and fluid fluxes through the tumor. The study of solid stress has also revealed that increased pressure in the TME, linked not only to tumor growth but also to the deposition of collagen I and hyaluronan, constrains blood vessels, hence not only further increasing interstitial fluid pressure but also preventing optimal delivery of anticancer drugs (Griffon-Etienne et al., 1999) (Figure 2B). This early observation illustrates the need to consider mechanical properties in cancer for the design of therapies.

## A Bright Future for 3D Cell Culture in Mechanomedicine

Mechanomedicine has been defined as the art of mechanobiology-based medicine (Wang, 2017). Thus, it may be applied to pathological conditions for which mechanobiology is either paramount for organ and tissue functions (e.g., cardiovascular, reproductive and respiratory systems), or maybe used to study and possibly target diseased tissues and cells, as it is the case in cancer (Ma et al., 2016; Özkale et al., 2021). Cancer mechanomedicine is illustrated for instance by research on modifying tumor vasculature to improve drug efficacy. Indeed, intratumor fluid pressure mediated by solid stress not only leads to hypoxia, hence promoting invasion, metastasis and treatment resistance, but it also prevents proper drug delivery, which also contributes to chemoresistance (Stylianopoulos et al., 2018) (Figure 2B).

A fundamental question to address with 3D cell culture is whether increased TME stiffness during cancer progression modifies the intratumor phenotypic heterogeneity that defines aggressiveness; indeed, we measured potential selective pressure in light of a higher apoptotic rate in breast tumors cultured in 3300 Pa compared to 2000 Pa (Figure 2C), confirming observations made previously of a link between high mechanical stress, measured in an agarose matrix with fluorescent microbeads, and apoptosis (Cheng et al., 2009). Moreover, we demonstrated that phenotypic intratumor heterogeneity occurred even when starting from a single cell to produce a tumor on top of an island of collagen I of 3300 Pa (Jain et al. 2020). One possible explanation for the induction of heterogeneity is the evolving force gradient within the tumor (Figure 2D). The importance of the impact of matrix stiffness on creating phenotypic heterogeneity is also supported by the fact that certain types of cancer cells, notably those involved in tumorigenesis require a soft microenvironment to proliferate (Liu et al., 2012).

Our current understanding of the impact of mechanotransduction on cancer phenotypes is limited to a correlation between TME and metastatic potential. Tensegrity and dynamic reciprocity models have brought enough incentives to consider that changes in stiffness within cells are also essential to study in order to fully develop mechanomedicine, especially since, opposite to the situation in the interstitium, invasive cells appear softer compared to nonmalignant and preinvasive cells; however, upon cancer progression cells acquire increased plasticity that might render them stiffer depending on external stimuli (Baker et al., 2010; Plodinec et al., 2012). Following incubation with anticancer drugs, treatment-resistant prostate cancer cells and leukemia cells display higher stiffness compared to untreated cells, as measured with AFM in 2D culture (Raudenska et al., 2019). If changes in intracellular stiffness control the sensitivity to anticancer drugs, further experiments will require 3D cell culture for validation, since such sensitivity is notoriously different between 2D and 3D cultures. It will also be necessary to identify targets of mechanostimuli responsible for resistance to treatment. For instance, shear stress applied to breast cancer cells with cell culture medium run through the ECM (agarose-collagen I) led to the activation of *PLAU* and linked this gene to increased resistance to paclitaxel (Novak et al. 2019). A wound healing system with compression with a rigid weight disc on an agar cushion on top of glioblastoma cells was used to mimic solid stress of cells detaching from the tumor within a confined skull. Results revealed a link between miR548 and increased migration as well as an influence on genes associated with chemoresistance (*TMEM45Q*) and angiogenesis (*CTGF*, *VEGFA, VEGFB*) (Calhoun et al., 2020). However, these results were obtained with different types of matrices, which makes it difficult to identify strict mechanical impact from a combination of mechanical and biochemical signaling. Further understanding of nuclear homeostasis in response to mechanical impact from well-characterized TME would strengthen the field of cancer mechanomedicine.

Another topic of importance for mechanotransduction to the cell nucleus that will influence mechanomedicine is tissue geometry. Physical constraints associated with a specific geometry were shown to control tissue morphogenesis, by locally influencing the concentration of morphogens in the microenvironment (Nelson et al., 2006), and the organization of the cell nucleus (Lelièvre and Chittiboyina, 2018). Regarding cancer, we have shown different levels of drug sensitivity for tumors depending on their location on flat vs. curved geometry (Vidi et al., 2014). Geometry-induced mechanotransduction to the cell nucleus has been clearly demonstrated in 2D culture on supports with defined geometry (Gomez et al., 2010). However, it is difficult to separate the effect of tissue geometry and that of matrix stiffness on cells cultured in 3D. Indeed, direct force application (which could be linked to matrix stiffness) influences tissue geometry and the composition of the ECM (Matsugaki et al., 2013; Muncie et al., 2020). Moreover, tumor proliferation and invasion are stimulated within a duct made of non-neoplastic epithelial cells only when mechanical stress is high, hence showing that mechanical stress acts independently on (or on top of) tissue geometry (Boghaert et al., 2012). Our preliminary studies with non-neoplastic breast epithelial cells suggest that a duct-like curved geometry modifies the effect of increasing matrix stiffness on cell phenotypes compared to increasing matrix stiffness on a flat geometry, which suggests that both physical aspects (geometry and matrix stiffness) have complementary impacts on phenotypes (Lelièvre laboratory, unpublished data).

In conclusion, the investigation of ECM-mediated mechanotransduction in a physiologically relevant context is crucial in furthering research aimed to overcome cancer progression and treatment resistance. In the above text, we have illustrated possibilities to induce intratumor phenotypic heterogeneity, a driver towards resistance. There is evidence that heterogeneity also exists in the cells’ capabilities to exert compressive stresses within a population (Mohagheghian et al., 2018). The 3D cell culture platforms will need to integrate different physical characteristics and physical stress measurement methods to best render the phenotypic heterogeneity of cancers. Actually, the TME is likely to contribute to the mixture of phenotypes because of the heterogeneity in matrix stiffness at the tumor periphery (Acerbi et al., 2015) and in fluid flow (Evje and Waldeland, 2019). Moreover, to properly tune ECM and fluid flows, tumor models should include stromal cells like fibroblasts that greatly contribute to cancer progression via their modulation of the TME.

## Data Availability

The raw data, from the Lelièvre laboratory, supporting the conclusions of this article will be made available by the authors, without undue reservation.
